# Pulsed electromagnetic fields inhibit mandibular bone deterioration depending on the Wnt3a/β-catenin signaling activation in type 2 diabetic db/db mice

**DOI:** 10.1038/s41598-022-10065-7

**Published:** 2022-05-04

**Authors:** Jianjun Li, Jing Cai, Liheng Liu, Yuwei Wu, Yan Chen

**Affiliations:** 1 Second Clinical Division, Peking University School and Hospital of Stomatology & National Clinical Research Center for Oral Diseases & National Engineering Laboratory for Digital and Material Technology of Stomatology & Beijing Key Laboratory of Digital Stomatology, 22 Zhongguancun South Avenue, Beijing, 100081 China; 2 College of Basic Medicine, Shaanxi University of Chinese Medicine, Xianyang, China; 3 Beijing Healya Technology Limited, Beijing, 100195 China; 4 Department of Obstetrics, Beijing Obstetrics and Gynecology Hospital, Capital Medical University, Beijing, China

**Keywords:** Bone, Metabolic bone disease

## Abstract

Type 2 diabetes mellitus (T2DM) patients have compromised mandibular bone architecture/quality, which markedly increase the risks of tooth loosening, tooth loss, and failure of dental implantation. However, it remains lacks effective and safe countermeasures against T2DM-related mandibular bone deterioration. Herein, we studied the effects of pulsed electromagnetic fields (PEMF) on mandibular bone microstructure/quality and relevant regulatory mechanisms in T2DM db/db mice. PEMF exposure (20 Gs, 15 Hz) for 12 weeks preserved trabecular bone architecture, increased cortical bone thickness, improved material properties and stimulated bone anabolism in mandibles of db/db mice. PEMF also upregulated the expression of canonical Wnt3a ligand (but not Wnt1 or Wnt5a) and its downstream β-catenin. PEMF improved the viability and differentiation of primary osteoblasts isolated from the db/db mouse mandible, and stimulated the specific activation of Wnt3a/β-catenin signaling. These positive effects of PEMF on mandibular osteoblasts of db/db mice were almost totally abolished after Wnt3a silencing in vitro, which were equivalent to the effects following blockade of canonical Wnt signaling using the broad-spectrum antagonist DKK1. Injection with Wnt3a siRNA abrogated the therapeutic effects of PEMF on mandibular bone quantity/quality and bone anabolism in db/db mice. Our study indicates that PEMF might become a non-invasive and safe treatment alternative resisting mandibular bone deterioration in T2DM patients, which is helpful for protecting teeth from loosening/loss and securing the dental implant stability.

## Introduction

Diabetes mellitus (DM) is a chronic metabolic disease with elevated blood glucose resulted from decline in either insulin production or insulin sensitivity. Over 450 million people were diagnosed with diabetes, and type 2 diabetes mellitus (T2DM) accounts for more than 90% of all DM cases worldwide^1^. T2DM can induce various chronic damage to the heart, kidneys, nerves, eyes as well as the skeleton. Patients with T2DM have compromised microarchitecture and mechanical properties in long bone, leading to significant increase in bone fragility^2,3^. It is reported the T2DM patients have significant increase in fracture risks in both long bone and vertebrae, and the extent of the increase depends on the skeleton site and severity of diabetes^4–6^. As the largest and strongest bone in the human skull supporting the mouth motion and housing the teeth, the mandibular bone’s structure/function is also altered in T2DM. Both clinical and experimental evidence suggests that mandibular bone microstructure and quality are deteriorated in T2DM as compared with controls^7,8^, which markedly increase the risks of tooth loosening, tooth loss, and failure of dental implantation^9,10^. However, it still lacks effective countermeasures against T2DM-related bone deterioration either in mandible or other skeletal sites (*e.g.*, spine and long bone). Furthermore, the mandible has big difference in the morphology and function with other skeletal sites, and the responses to anabolic agents or catabolic stimuli are also different^11,12^. Thus, it is necessary to develop effective osteoprotective approaches specific for mandibular bone in T2DM patients.

As a non-invasive and easy biophysical approach, treatment with low-intensity and low-frequency pulsed electromagnetic fields (PEMF) has proven effective against a wide range of bone diseases. The earliest studies have shown that PEMF exposure can significantly accelerate fresh fracture healing and promote non-union fracture repair in clinics^13^. Subsequent studies have demonstrated that PEMF treatment stimulates bone osseointegration and osteogenesis of various types of implants (e.g., titanium, stainless steel and polymeric materials)^14–16^. Over the past decade, growing evidence suggests that PEMF exposure significantly increases bone mass and strength in normal animals and osteoporotic animals induced by ovariectomy and disuse^17–21^. Some clinical trials confirm the therapeutic effects of PEMF on osteoporosis in postmenopausal women^22–24^. Although the mechanism by which PEMF regulates bone metabolism remains not fully clarified, substantial evidence reveals that the osteoblast is potentially the major cell type for detecting external PEMF signals^20,25,26^. The PEMF signal is received at the cell membrane of osteoblasts and then initiates a cascade of intracellular signaling events, and thereby modulates osteogenesis-related gene expression (e.g., Runx2, β-catenin)^20,26^. Some recent in vivo studies have shown that PEMF treatment is also able to resist DM-induced mineral loss of long bone^27–29^. However, whether PEMF can effectively resist diabetic mandibular bone deterioration, especially for more prevalent T2DM individuals, remains poorly understood.

The canonical Wnt signaling (Wnt/β-catenin signaling) pathway is crucial for osteoblast development and proper bone formation^30^. The canonical Wnt signaling is activated via ligation of extracellular Wnts to Frizzled and Lrp5/6 co-receptors on plasma membrane and subsequent induction of intracellular β-catenin accumulation. It has been shown that active canonical Wnt signaling augments mandibular bone mass/architecture, and its inhibition results in the decrease of mandibular bone formation^31,32^, highlighting the essential role of Wnt/β-catenin signaling in the maintenance of mandibular bone hemostasis. In addition, the genetically diabetic db/db mouse is a well-accepted T2DM model characterized by hyperphagia, obesity, hyperglycemia, and hyperinsulinemia due to the deficiency of leptin receptor^33^. The db/db mouse has been proved to exhibit osteoporotic phenotype, which is possibly associated with decreased bone formation and increased bone resorption^34,35^. Therefore, in the current study, we aim to (i) identify the effects of PEMF exposure on mandibular bone mass, microarchitecture, material properties, and bone turnover in T2DM db/db mice; (ii) clarify the mechanism by which PEMF exposure regulates mandibular bone metabolism in db/db mice via both in vitro and in vivo Wnt silencing experiments.

## Materials and methods

### Animals studies

We purchased 12-week-old male db/db mice on the C57BKS background (BKS.Cg-*m*+*/*+*Lepr*^*db*^/J) and their homozygous littermate wild-type control (WT) mice from the Jackson Laboratory (Bar Harbor, Maine). The db/db mice with fasting blood glucose > 16.7 mmol/L was considered as qualified animal models. Mice were randomly assigned into three groups: the WT mice group (WT, *n* = 9), the db/db mice group (db/db, *n* = 9), and db/db mice with PEMF exposure group (db/db + PEMF, *n* = 9). The db/db mice in the db/db + PEMF group were exposed to PEMF with 2 h/day for 12 weeks. All animals received two intramuscular injections of 8 mg/kg calcein (Sigma-Aldrich) on 14 and 4 days before sacrifice, respectively. For the in vivo Wnt3a inhibition experiment, 12-week-old male db/db mice were randomly distributed into four groups: db/db mice treated with control siRNA, db/db mice treated with Wnt3a siRNA, db/db mice treated with PEMF (2 h/day, 12 weeks) and control siRNA, and db/db mice treated with PEMF and Wnt3a siRNA. The Wnt3a siRNA or control RNA was prepared with Entranster™ in vivo Transfection Reagent, and then injected subcutaneously over the mandibular surface of mice once per day at the dosage of 40 μL. At the end of experiment, animals were euthanized using carbon dioxide inhalation, followed by confirmation of death with cervical dislocation. Left mandible were harvested for micro-computed tomography (micro-CT), histomorphometric and biomechanical analyses. Right mandibles were harvested and sectioned into two halves, one was immersed in fixed in 4% paraformaldehyde for static bone histomorphometric analysis, and the other was stored in liquid nitrogen for qRT-PCR analysis. All animal procedures were performed according to the Guideline for the Care and Use of Laboratory Animals and with the ethical approval from the Laboratory Animal Ethics and Welfare Committee of the Peking University. All experiments were performed in compliance with the ARRIVE guidelines, and all methods were strictly carried out following the relevant guidelines and regulations.

### PEMF exposure

The PEMF system and waveform were introduced in detail in our previous study^28,29^. In brief, the electromagnetic fields were generated by a Helmholtz coil assembly with three coaxial coils (80 cm diameter, 30.4 cm apart from each other) which was connected with a signal generator. The turns of the central coil and outside coils were 266 and 500, respectively. This coil model has higher uniformity of electromagnetic fields than the traditional two-coil model^21^. The PEMF waveform produced by the signal generator was a pulsed burst (burst width, 5 ms; pulse width, 0.2 ms; pulse wait, 0.02 ms; burst wait, 60 ms; pulse rise, 0.3 μs; pulse fall, 2.0 μs) repeated at 15 Hz, which has shown significant therapeutic effects on bone loss in ovariectimized and tail-suspended animals^20,21^. Mice were housed in non-magnetic cages, and the cage bottom of the plastic mouse cage was aligned with the center of the coils. The peak magnetic field of the coils was calculated to be approximately 2.0 mT using an oscilloscope (Agilent Technologies, Santa Clara, CA). To examine the induced electric fields, an electrical potential detecting coil (5 cm diameter, 20 turns) was placed in the midcenter of the Helmholtz coils with the coil parallel to the Helmholtz coils. The induced peak electrical field was determined to be ~ 2 mV/cm.

### Micro-CT

A desktop micro-CT system was used to evaluate mandibular bone microarchitecture (GE healthcare, Madison, WI). At sacrifice, the mandibular samples were dehydrated in 80% ethanol for 2 days, and then scanned at a resolution of 8 μm, a voltage of 80 kV, and a current of 80 μA. After scanning, transverse micro-CT slices were reconstructed to the 3-dimentional image. A volume of interest (VOI), excluding the molar and incisor teeth as well as cortical bone, was selected to evaluate trabecular bone microarchitecture. The trabecular bone and cortical bone were distinguished according to the gray scale value (the threshold was set as 75 units) in the micro-CT image. The indices of trabecular bone volume per tissue volume (BV/TV), trabecular number (Tb.N), trabecular thickness (Tb.Th), and trabecular separation (Tb.Sp), and cortical thickness (Ct.Th) were quantified to analyze trabecular and cortical bone microarchitecture.

### Bone histomorphometry

Following embedding in methylmethacrylate (MMA), mandibular samples were cut along the sagittal plane using a Leica 2500E non-decalcification sliding microtome to a thickness of about 50 μm. Double-labeled images were capture using a Leica DMLA fluorescence microscope. For dynamic bone histomorphometric parameter analysis, the mineral apposition rate (MAR, the mean spacing between the fluorescent markers divided by the 10-day interlabel period) and bone formation rate (BFR/BS, defined as MAR*MS/BS, MS/BS was calculated as the sum of the double-labeled perimeter and one-half of the single-labeled perimeter per bone surface) were measured, respectively. Following fluorescence imaging, mandibular samples were stained with hematoxylin and eosin (H&E) to further analyze the histology of mandibular cancellous bone, and the trabecular bone area per total area was measured based on the H&E images. For static bone histomorphometric analyses, the fixed mandibular specimens with PFA were immersed in decalcifying reagents including 10% ethylenediamine tetraacetic acid (EDTA, pH: 7.4) for 3–4 weeks. Paraffin-embedded tissue sections with a thickness of 5 μm were stained with toluidine blue to identify osteoblasts, and the number of osteoblasts per unit of trabecular bone surface (N.OB/BS) was determined. The number of osteoclasts per unit of trabecular bone surface (N.OC/BS) was calculated based on tartrate resistant acid phosphatase (TRAP) staining to characterize osteoclasts.

### Nanoindentation

The mandibular samples embedded in MMA were then sectioned into 2 mm slices using a low-speed saw (Leica 2500E, Leica SpA, Milan, Italy). The surfaces of the specimens were ground using silicon carbide abrasive papers with progressively finer grit (800, 1000 and 1200), and then rehydrated in saline solution for 24 h. Nanoindentation was conducted using an Agilent G200 indenter (Agilent Technologies Inc., Chandler, AZ, USA) equipped with a Berkovich diamond tip. Arrays (3 × 3 indents) were created in mandibular cancellous bone in each specimen with 50 μm spacing, and the results were then averaged. The indenter was loaded into the specimen to a maximum depth of 4000 nm at a constant strain rate with 0.05 s^-1^, followed by 10 s holding at the peak load to minimize the viscoelastic behavior. Unloading was then given to 10% of the peak load at the maximum loading rate followed by 60 s holding for calculating the thermal drift. The indenter was finally removed from the sample surface. The indices of intrinsic material properties, including elastic modulus and contact hardness were calculated according to the force–displacement curve^36^.

### RNA isolation and qRT-PCR

Right mandibles (frozen in liquid nitrogen) were ground with a pestle in a sterile mortar containing liquid nitrogen to obtain a fine powder. A kit for total RNA isolation was used in accordance with the manufacturer's protocols (Invitrogen, Carlsbad, CA). RNA was reverse transcribed using SuperScript III reverse transcriptase. Real-time reaction was conducted using the SYBR Green on the ABI 7300 PCR system. The relative mRNA expression levels of each target gene were normalized to the house-keeping gene β-actin. The measurements were carried out in triplicate and calculated using the 2^-ΔΔCt^ approach. Primers and probes utilized in the present study were depicted as follows: GTGTGAGCTTAACCCTGC (forward) and ACAGGGAGGATCAAGTCC (reverse) for OCN, TGCACCTACCAGCCTCACCATAC (forward) and GACAGCGACTTCATTCGACTTCC (reverse) for Runx2, GAAAGGAGGCACAAAGAAG (forward) and CACCAAGGAGTAGGTGTGTT (reverse) for Osx, AGAAAAGCAACAGAAGCC (forward) and GACCGCAGTCCGTCTAAG (reverse) for BMP2, TTGCGACCATTGTTAGCCACATA (forward) and TCAGATCCATAGTGAAACCGCAAG (reverse) for TRAP, CAGCAGAACGGAGGCATTGA (forward) and CTTTGCCGTGGCGTTATACATACA (reverse) for Cathepsin K, ACCAAAGTGAATGCCGAGAGAG (forward) and ACGCTGCTTTCACAGAGGTC (reverse) for OPG, GGGGAGGCAACTGTCACCTT (forward) and TAGTCTGTAGGTACGCTTCC (reverse) for RANKL, GGAATGATGCCACAGAGGTC (forward) and CTGTCAGGAAGCGGGTGTAG (reverse) for Sost, GGACTTGCTTCTCTTCTCATAGCC (forward) and CCACACAGGCATAGAGTGTCTGC (reverse) for Wnt1, CTCCTCTGCAGCCTGAAGC (forward) and GTGGACGGTGGTGCAGTT (reverse) for Wnt3a, CTGCGGAGACAACATCGACTA (forward) and CGTGGATTCGTTCCCTTTCTCTA (reverse) for Wnt5a, CAGCACCACAGGCCACCAA (forward) and TCGAGACATTCCTGGAAGAG (reverse) for Lrp6, GGAAAGCAAGCTCATCATTCT (forward) and AGTGCCTGCATCCCACCA (reverse) for β-catenin, and GCCAACACAGTGCTGTCT (forward) and AGGAGCAATGATCTTGATCTT (reverse) for β-actin.

### Isolation and culture of primary osteoblasts

Primary osteoblasts from 12-week-old male db/db mice or their homozygous littermate WT mice were isolated from mandibular bone in sterile conditions using the enzyme digestion method according to the similar protocols as described previously^37–39^. In brief, after removal of muscle, soft tissues, teeth and periosteum, bone chips of mandibles were cut into 1–2 mm^3^ pieces and then digested by 0.25% trypsinase containing 0.02% EDTA for 25 min followed by incubation in 5 ml Hanks solution containing 0.1% collagenase I and 0.05% trypsin containing 0.004% EDTA for 90 min. The supernatant was centrifuged to collect the released cells. Then, cells were cultured in α-MEM with 10% foetal bovine serum (FBS) and 1% penicillin–streptomycin. The osteoblast phenotype was verified via Alkaline phosphatase (ALP) and Alizarin red S staining. For the inhibitor experiment, the cell culture medium was supplemented with the Wnt/β-catenin signaling blocker DKK1 (1 µg/mL; Amgen, Millstadt, IL). For the gene silencing experiment, siRNA for mouse Wnt3a with scrambled sequences was obtained from Santa Cruz (sc-41109), and 0.1 μM siRNA was added into primary osteoblasts using the Lipofectamine 3000 (Invitrogen). The transfection efficiency was examined by green fluorescent protein and the mRNA expression.

### Cell viability analysis and cytoskeleton staining

Cells in the PEMF group were subjected to PEMF exposure (2 h/day) at 37 °C for 3 days. For cell viability analysis, primary osteoblasts were incubated with 80 μL 3-(4,5-dimethylthiazol-2-yl)-2,5-diphenyltetrazolium bromide (MTT; Sigma) at 37 °C for 4 h, and then 800 μL DMSO was added to dissolve the formazan formed by MTT. The mixture was transferred to a multimode microplate reader (Tecan GENios, San Jose, CA, USA) to examine the optical density at 490 nm. For cytoskeleton structure analysis, primary osteoblasts were fixed in 4% paraformaldehyde for 5 min, permeabilized with 0.1% Triton X-100, and then stained with 50 mg/mL FITC (Sigma) for 40 min and DAPI (Sigma) for 10 min. Cells were then imaged under a confocal microscope (FV1000, Olympus, Tokyo, Japan) in five random fields of view.

### ALP staining and Alizarin red S staining

Primary osteoblasts were grown in osteogenic differentiation medium (α-MEM with 10% FBS, 1% penicillin–streptomycin, 50 µM ascorbic acid, and 10 mM β-glycerophosphate). Cells in the PEMF group were subjected to PEMF exposure (2 h/day) at 37 °C for 7 days. For ALP staining, cells were fixed in 4% formaldehyde for 10 min, and ALP staining was performed using the BCIP/NBT ALP color development kit. Then, specimens were imaged under an optical microscope (LEICA DM LA, Leica Microsystems, Heidelberg, Germany) in five random fields of view. For Alizarin red S staining, cells were fixed in 4% formaldehyde, and calcium deposits were then stained with 2% Alizarin Red S (pH = 8.3) followed by solubilization using 0.5 mL of 5% SDS in 0.5 N HCl for 30 min. The absorbance was then examined using a microplate reader at 405 nm (TECAN Infinite M200 Pro, TECAN, Switzerland).

### Western blotting

The procedures of protein extraction and western blotting were described as in details in our previous study^29^. In brief, cells in the PEMF group were exposed to PEMF at 37 °C for 3 days (2 h/day), and then protein lysates were prepared were prepared using RIPA buffer supplemented with 1 mM PMSF on ice. After quantification, protein extracts in equal amounts were loaded on a 10% Tris–glycine SDS-PAGE gel, and then electrotransferred to PVDF membranes. The blots were blocked in 5% bovine serum albumin (BSA) for 1 h, followed by incubation with primary antibodies against OCN (Abcam, Cambridge, MA), Runx2 (Biorbyt Ltd., Cambridge, UK), Wnt3a (Novus Biologicals, Littleton, CO), p-GSK-3β (Abcam), β-catenin (EMD Millipore, Billerica, MA) and β-tubulin (Bioworld technology, Inc., Louis Park, MN) in TBST containing 5% BSA overnight at 4 °C. β-tubulin was employed as the protein loading control. After incubation with a 1:3000 dilution of HRP-conjugated secondary antibody for 1 h at room temperature, the membranes were visualized using the ECL chemiluminescence system (GE ImageQuant 350, GE Healthcare). The Quantity One Software (Bio-Rad) was used to quantify the densities of the bands.

### Statistical analysis

All data were represented as the mean ± standard error of mean (SEM). Statistical computations were performed using SPSS 21.0 software (SPSS, Chicago, IL). The Kolmogorov–Smirnov test was employed to evaluate the normal distribution of data, and the Levene's test was used to analyze the homogeneity of variance. Each specific parameter was observed to obey normal distribution and homoscedasticity. Statistical significance between each two groups was determined using one way analysis of variance (ANOVA) followed by a Bonferroni’s post-hoc multiple comparison. *P* < 0.05 was accepted as statistically significant.

## Results

### PEMF improves mandibular bone architecture and material properties in db/db mice

**Figure 1:**
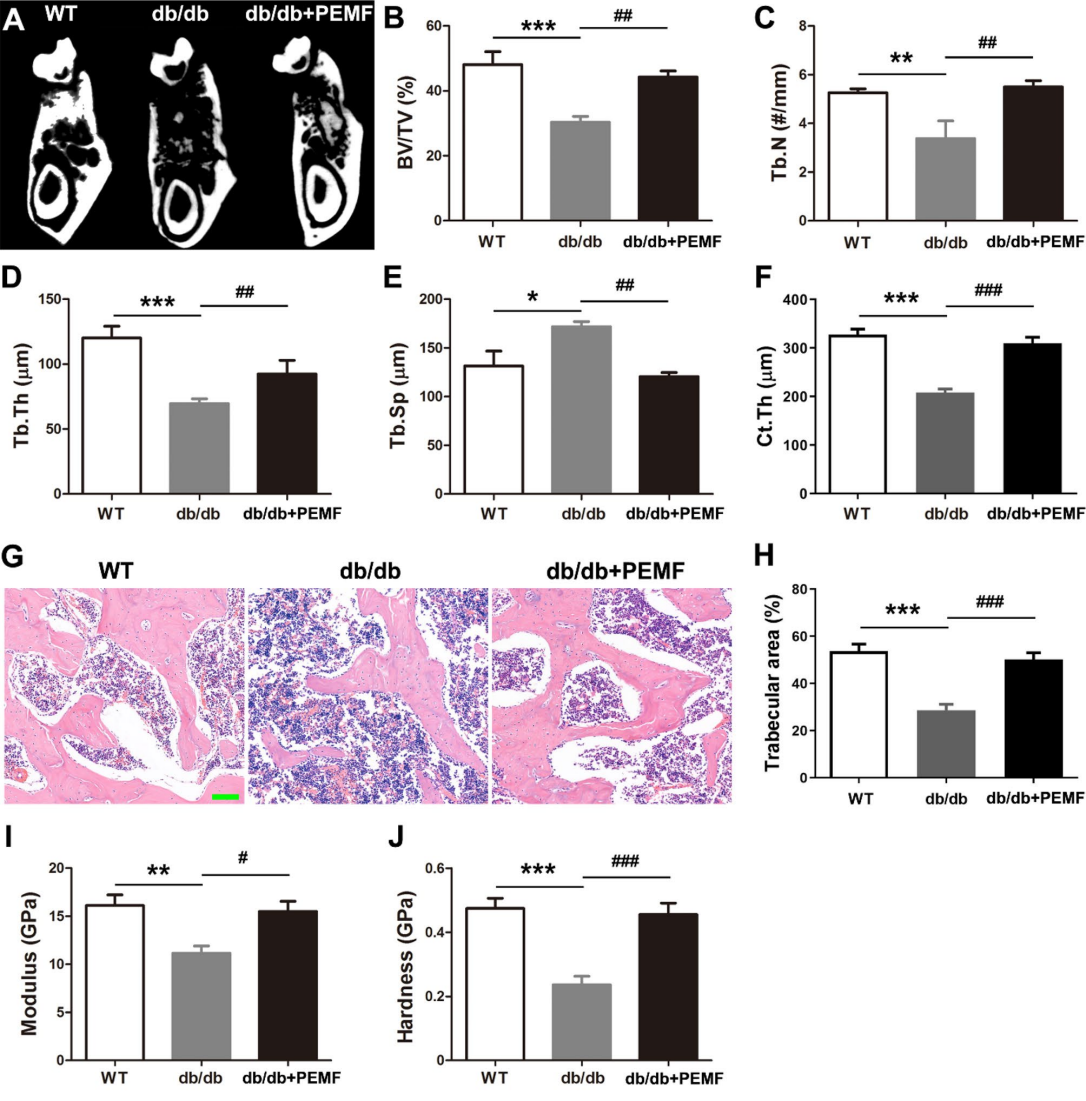
Pulsed electromagnetic fields (PEMF) exposure for 12 weeks improves in vivo mandibular bone microarchitecture and material properties in type 2 diabetic db/db mice. (**A**) Representative micro-CT images showing mandibular bone microarchitecture in the wild-type mice group (WT), the db/db mice group (db/db), and the db/db mice with PEMF stimulation group (db/db + PEMF). **(B–F)** Statistical comparison of micro-CT data, including **(B)** trabecular bone volume per tissue volume (BV/TV), **(C)** trabecular number (Tb.N), **(D)** trabecular thickness (Tb.Th), **(E)** trabecular separation (Tb.Sp), and **(F)** cortical thickness (Ct.Th). **(G,H)** Analysis of mandibular trabecular bone microstructure based on H&E staining. Scale bar represents 100 µm. **(I,J)** Analysis of mandibular trabecular bone material properties based on nanoindentation testing, including **(I)** modulus and **(J)** hardness. Data are represented as mean ± SEM (*n* = 9/group). *P < 0.05, **P < 0.01, ***P < 0.001 vs. the WT group; ^#^*P* < 0.05, ^##^*P* < 0.01, ^###^*P* < 0.001 vs. the db/db group by one way analysis of variance (ANOVA) followed by Bonferroni’s multiple comparison.

In contrast to the WT group, mice in the db/db group exhibited compromised cancellous and cortical bone architecture in the mandible according to the micro-CT imaging analysis, as evidenced by significantly decreased BV/TV, Tb.N, Tb.Th and Ct.Th, and increased Tb.Sp (Fig. 1A–F, P < 0.05). Furthermore, mice in the db/db + PEMF group (the db/db mice with PEMF exposure for 12 weeks) had significantly higher BV/TV, Tb.N, Tb.Th, Ct.Th and lower Tb.Sp than the db/db group (Fig. 1A–F, P < 0.01). However, no difference was observed in all the micro-CT indices between the db/db group and db/db + PEMF group. These results demonstrate that the deterioration in cancellous and cortical bone architecture observed in the mandible of db/db mice was inhibited by PEMF exposure. Moreover, the H&E staining assays of bone sections reveal that mandibular trabecular area in the db/db + PEMF group was significantly higher than that in the db/db group (Fig. 1G,H, P < 0.001). According to the nanoindentation results, the indices of mandibular bone material properties (including modulus and hardness) in the db/db group were significantly lower than those in the WT group (Fig. 1I,J, P < 0.01). We found that PEMF stimulation for 12 weeks significantly increased the modulus and hardness in db/db mice (Fig. 1I,J, P < 0.05), suggesting that PEMF exposure improves mandibular bone material properties in db/db mice in addition to bone’s microarchitecture.

### PEMF induces anabolic effects in the mandible of db/db mice

**Figure 2:**
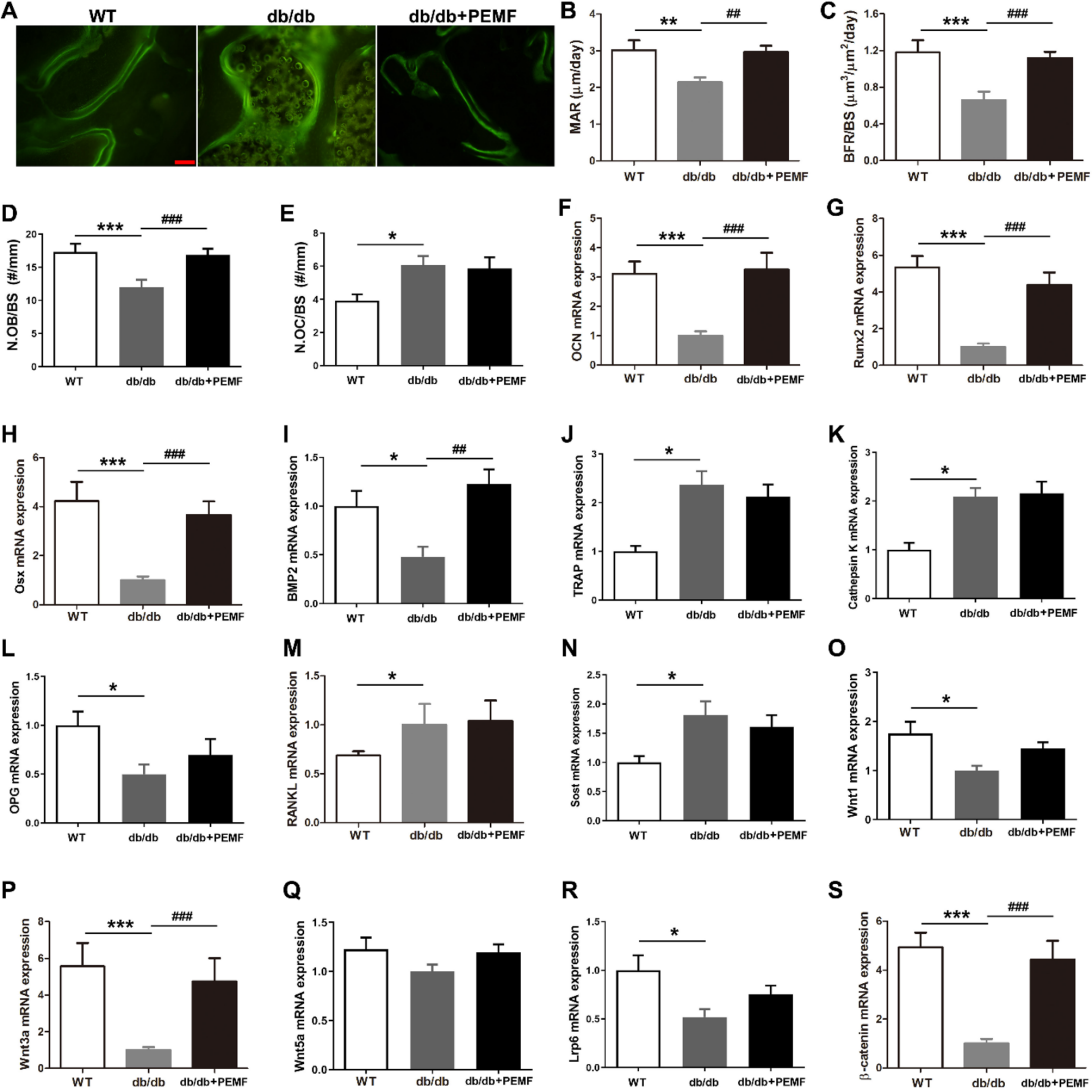
Pulsed electromagnetic fields (PEMF) exposure for 12 weeks increases in vivo bone formation, up-regulates the expression of osteoblastic differentiation-related genes, and stimulates the expression of canonical Wnt signaling genes in mandibular bone of type 2 diabetic db/db mice. (**A**) Representative calcein double-labeling images in mandibular trabecular bone surfaces. Scale bar represents 100 µm. **(B,C)** Statistical comparison of calcein double-labeling data, including **(B)** mineral apposition rate (MAR) and **(C)** bone formation rate per bone surface (BFR/BS). **(D,E)** Static bone histomorphometry to quantify osteoblast numbers per millimeter of trabecular bone surface (N.OB/BS) and osteoclast numbers per millimeter of trabecular bone surface (N.OC/BS) in mandibular specimens. **(F–N)** Analysis of mandibular bone gene expression associated with osteoblastic differentiation (including osteocalcin, Runx2, Osx and BMP2), osteoclastogenesis (TRAP, Cathepsin K, OPG and RANKL) and osteocyte-specific marker (Sost). **(O–S)** Analysis of mandibular bone gene expression of canonical Wnt1 and Wnt3a ligands, non-canonical Wnt5a ligand, transmembrane protein Lrp6, and downstream β-catenin of canonical Wnt signaling. Data are represented as mean ± SEM (*n* = 9/group). *P < 0.05, **P < 0.01, ***P < 0.001 vs. the WT group; ^##^*P* < 0.01, ^###^*P* < 0.001 vs. the db/db group by one way analysis of variance (ANOVA) followed by Bonferroni’s multiple comparison.

Based on calcein double-labeling results, mice in the db/db group were found to exhibit lower mandibular bone MAR and BFR/BS than the WT group (Fig. 2A–C, P < 0.01), revealing compromised bone formation in db/db mice. The db/db mice exhibited lower osteoblast number and higher osteoclast number on mandibular trabecular bone surface than the WT mice (Fig. 2D,E, P < 0.05). The db/db group also had decreased expression of osteoblastic differentiation-related genes (including OCN, Runx2, Osx and BMP2) and increased expression of osteoclast-related genes (including TRAP, Cathepsin K and RANKL/OPG) in mandibular bone as compared with the WT group (Fig. 2F–M, P < 0.05). The db/db mice also had higher osteocyte-specific Sost gene expression than the WT mice (Fig. 2N, P < 0.05). Mice in the db/db + PEMF group exhibited significantly higher mandibular bone MAR, BFR/BS and N.OB/BS than the db/db group (Fig. 2A–D, P < 0.01), and also had higher OCN, Runx2, Osx and BMP2 gene expression in the mandible than the db/db group (Fig. 2F–I, P < 0.001). However, PEMF did not cause change in N.OC/BS (Fig. 2E) or osteoclastogenesis-related TRAP, Cathepsin K, OPG or RANKL gene expression (Fig. 2J–M). PEMF exposure also had no impact on mandibular Sost gene expression in db/db mice (Fig. 2N). Furthermore, mice in the db/db group showed lower Wnt1 and Wnt3a expression in the mandible than the WT group (Fig. 2O,P, *P* < 0.05). PEMF significantly increased Wnt3a and β-catenin gene expression in the mandible in db/db mice, but did not change Wnt1, Wnt5a or Lrp6 expression (Fig. 2O–S).

### PEMF improves the viability and differentiation of osteoblasts in vitro from db/db mouse mandible

**Figure 3:**
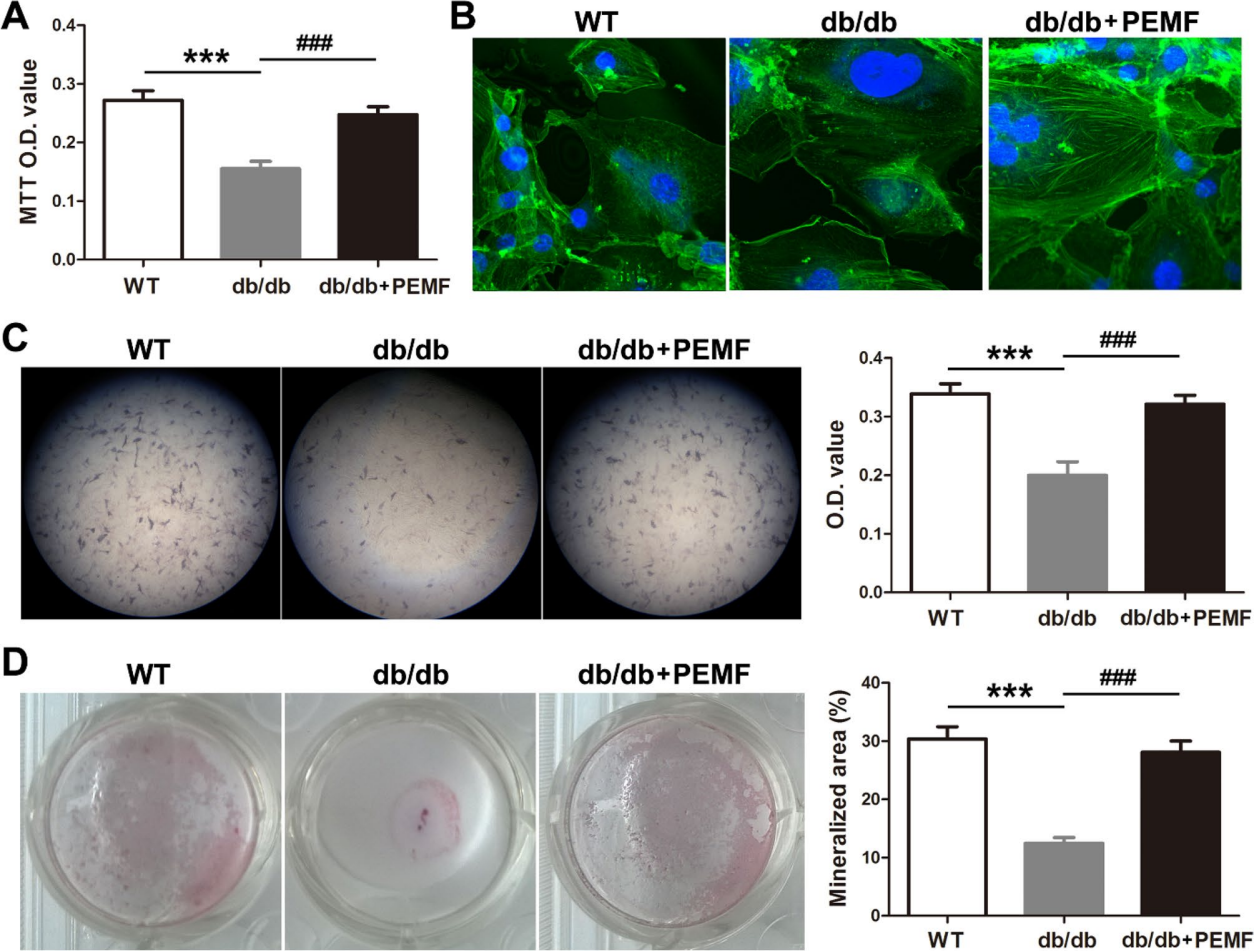
Pulsed electromagnetic fields (PEMF) exposure improves in vitro osteoblast viability and accelerates osteoblast differentiation in primary osteoblasts isolated from mandibular bone of type 2 diabetic db/db mice. (**A**) MTT assays for in vitro primary osteoblast proliferation. (**B**) F-actin cytoskeleton imaging of in vitro primary osteoblasts based on FITC-labeled phalloidin staining. (**C**) In vitro osteoblast differentiation assays based on alkaline phosphatase staining. (**D**) In vitro osteoblast mineralization assays based on Alizarin red staining of the mineralized nodule. Data are represented as mean ± SEM (*n* = 7–10/group). ***P < 0.001 vs. the WT group; ^###^*P* < 0.001 vs. the db/db group by one way analysis of variance (ANOVA) followed by Bonferroni’s multiple comparison.

Similar with the in vivo results, primary osteoblasts isolated from db/db mouse mandible exhibited decreased cellular proliferation as compared with the WT mice (Fig. 3A, P < 0.001). PEMF significantly enhanced cellular proliferation in primary osteoblasts isolated from db/db mouse mandible (Fig. 3A, P < 0.001), and also induced visible increase in the number and thickness of F-Actin microfilament (Fig. 3B). Further, according to the alkaline phosphatase and mineralized nodule staining, we found that osteoblasts isolated from db/db mouse mandible showed reduced cellular differentiation and mineralization than those isolated from the WT mice (Fig. 3C,D, *P* < 0.001). PEMF exposure significantly improved the differentiation and mineralization in primary osteoblasts isolated from db/db mouse mandible (Fig. 3C,D, *P* < 0.001).

### PEMF stimulates the Wnt3a/β-catenin signaling activation in primary osteoblasts from db/db mouse mandible

**Figure 4:**
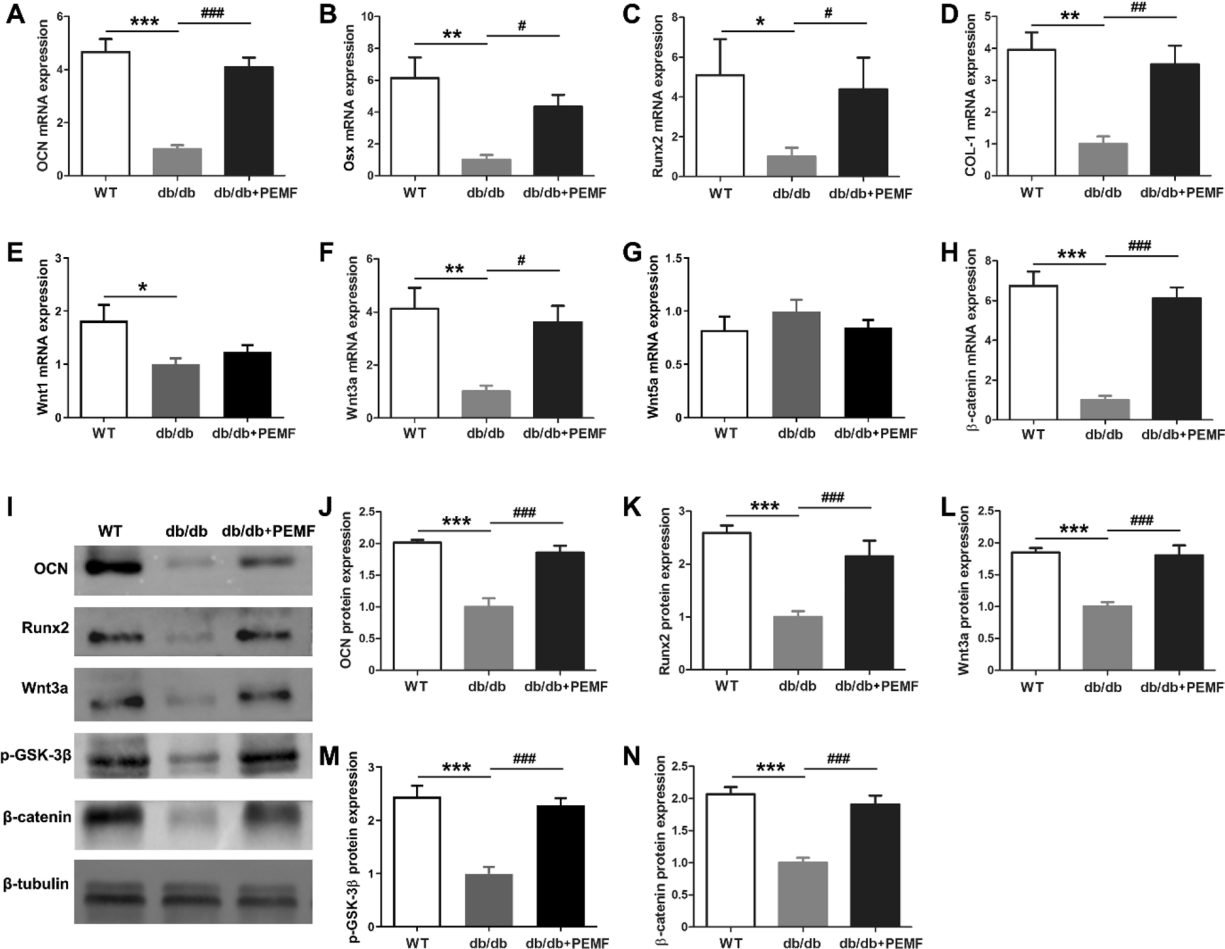
Pulsed electromagnetic fields (PEMF) exposure increases the expression of osteoblastic differentiation markers and stimulates the activation of Wnt3a/β-catenin signaling in primary osteoblasts in vitro isolated from mandibular bone of db/db mice. (**A–D**) Analysis of in vitro osteoblastic differentiation-related gene expression, including osteocalcin (OCN), Runx2, Osx and COL-1. **(E–H)** Analysis of in vitro gene expression of canonical Wnt1 and Wnt3a ligands, non-canonical Wnt5a ligand, and downstream β-catenin of canonical Wnt signaling in primary osteoblasts. **(I–N)** In vitro protein expression analysis of OCN, Runx2, Wnt3a, p-GSK-3β and β-catenin in primary osteoblasts based on western blotting assays. Data are represented as mean ± SEM (*n* = 7–10/group). *P < 0.05, **P < 0.01, ***P < 0.001 vs. the WT group; ^#^*P* < 0.05, ^##^*P* < 0.01, ^###^*P* < 0.001 vs. the db/db group by one way analysis of variance (ANOVA) followed by Bonferroni’s multiple comparison.

In contrast to primary osteoblasts from the WT group, the db/db group had significantly decreased expression of osteoblastic differentiation-related genes, including OCN, Osx, Runx2 and COL-1 (Fig. 4A–D, P < 0.05). PEMF significantly increased the gene expression of osteoblastic differentiation-related markers (Fig. 4A–D, P < 0.05). PEMF up-regulated the gene expression of Wnt3a and its downstream β-catenin, but did not change the gene expression of Wnt1a and Wnt5a (Fig. 4E–H). Similarly, our western blotting results demonstrate that PEMF stimulated the protein expression of osteoblastic differentiation-related OCN and Runx2 (Fig. 4I–K, P < 0.001). PEMF also significantly up-regulated the protein expression of Wnt3a, p-GSK-3β and β-catenin (Fig. 4L–N, P < 0.001), suggesting that PEMF exposure induced the activation of Wnt3a/β-catenin signaling in mandibular osteoblasts of db/db mice.

### Wnt antagonist or Wnt3a silencing blunts PEMF-induced acceleration of differentiation and proliferation of primary osteoblasts in db/db mouse mandible

**Figure 5:**
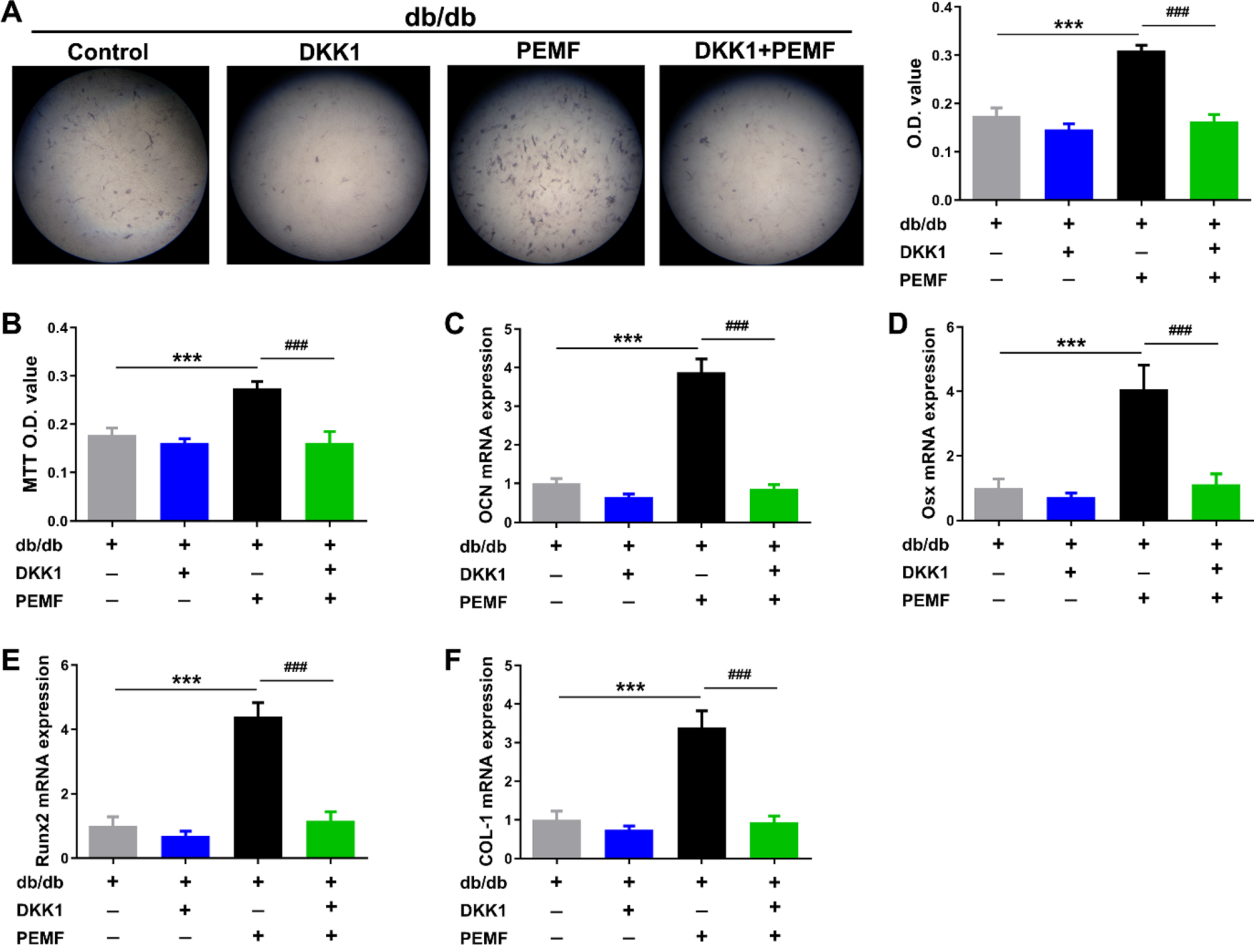
Wnt antagonist DKK1 blunts pulsed electromagnetic fields (PEMF)-induced acceleration of cellular differentiation and proliferation of in vitro primary osteoblasts isolated from mandibular bone of db/db mice. (**A**) In vitro osteoblast differentiation assays based on alkaline phosphatase staining. (**B**) MTT assays for in vitro primary osteoblast proliferation. (**C–F**) Analysis of in vitro osteoblastic differentiation-related gene expression, including osteocalcin (OCN), Runx2, Osx and COL-1. Data are represented as mean ± SEM (*n* = 7–10/group). ***P < 0.001 vs. the db/db mice group; ^###^*P* < 0.001 vs. the db/db mice with PEMF exposure group by one way analysis of variance (ANOVA) followed by Bonferroni’s multiple comparison.

**Figure 6:**
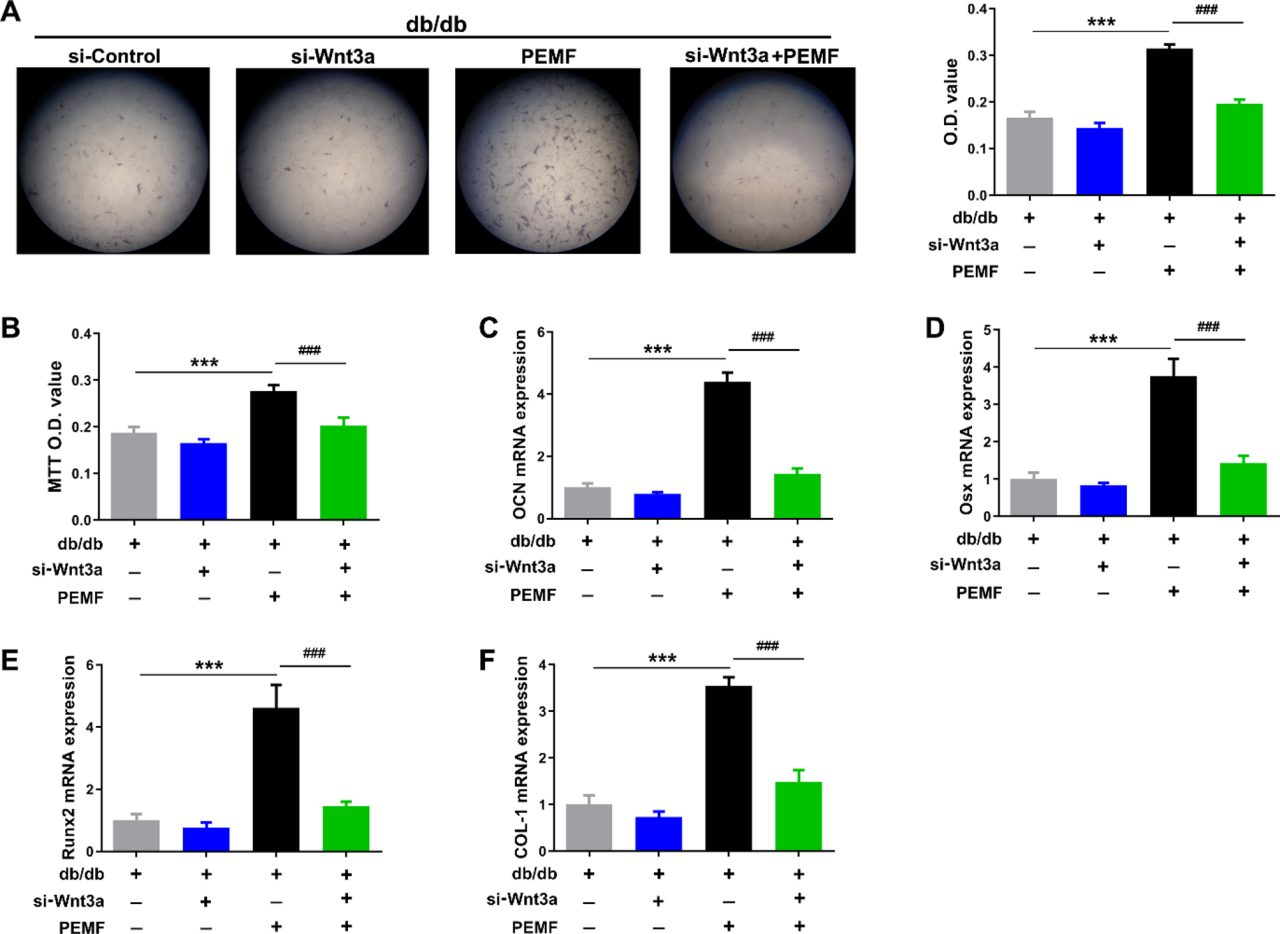
Pulsed electromagnetic fields (PEMF)-induced improvement in cellular differentiation and proliferation of in vitro osteoblasts isolated from mandibular bone of db/db mice is inhibited after Wnt3a silencing. (**A**) Osteoblast differentiation assays based on alkaline phosphatase staining. (**B**) MTT assays for primary osteoblast proliferation. (**C–F**) Analysis of osteoblastic differentiation-related gene expression, including osteocalcin (OCN), Runx2, Osx and COL-1. Data are represented as mean ± SEM (*n* = 7–10/group). ***P < 0.001 vs. the db/db mice group; ^###^*P* < 0.001 vs. the db/db mice with PEMF exposure group by one way analysis of variance (ANOVA) followed by Bonferroni’s multiple comparison.

The primary osteoblasts isolated from db/db mice treated with Wnt antagonist DKK1 exhibited trend with minor decrease in cellular proliferation (MTT assays) and differentiation (alkaline phosphatase staining and differentiation-related gene expression) as compared with the osteoblasts from db/db mice without DKK1 treatment, but these changes did not reach statistical significance (Fig. 5A–F). As expected, the primary osteoblasts from db/db mice exposed to PEMF had significantly higher proliferation and differentiation than those without PEMF stimulation (Fig. 5A–F, P < 0.001). In contrast to the db/db mouse osteoblasts with PEMF exposure, cells isolated from db/db mice treated with DKK1 together with PEMF had significantly decreased cellular proliferation and differentiation potential, according to the MTT assays, alkaline phosphatase staining and differentiation-related gene expression analysis (Fig. 5A–F, P < 0.001). Moreover, in comparison with the primary db/db osteoblasts with PEMF exposure, osteoblasts isolated from db/db mice with PEMF exposure and specific silencing of the Wnt3a subtype exhibited significant decrease in osteoblast proliferation and differentiation (Fig. 6A–F, P < 0.001), which was equivalent to the effects of broad-spectrum Wnt/β-catenin antagonist DKK1.

### Wnt3a silencing blunts PEMF-induced improvement in mandibular bone microarchitecture and bone formation in db/db mice

**Figure 7:**
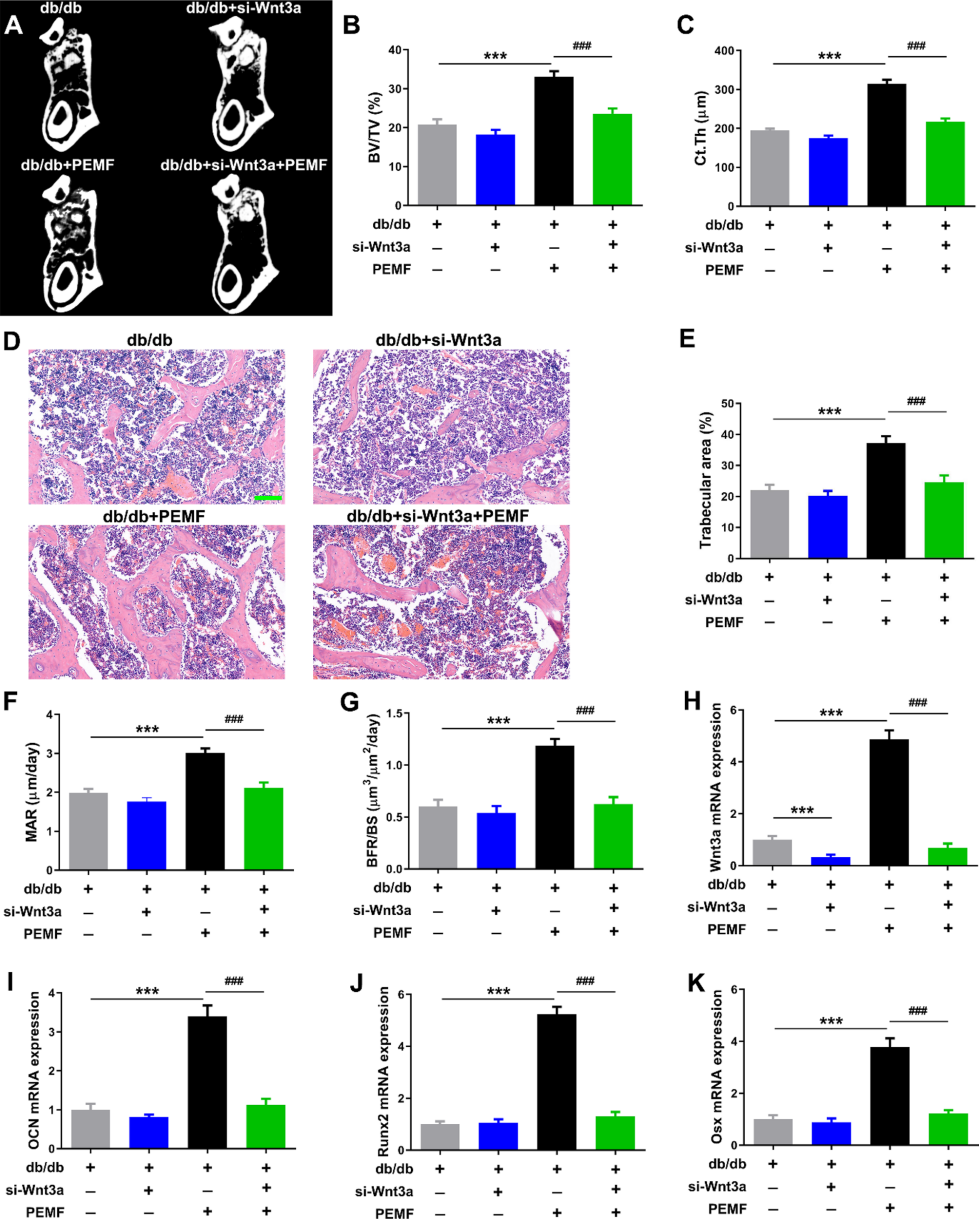
Wnt3a silencing blunts pulsed electromagnetic fields (PEMF)-induced improvement in mandibular bone microarchitecture, bone formation and osteoblastic activity in db/db mice in vivo. (**A–C**) Analysis of mandibular bone microarchitecture based on micro-CT imaging. **(D,E)** Analysis of mandibular trabecular bone microstructure based on H&E staining. Scale bar represents 100 µm. (**F,G**) Analysis of mandibular trabecular bone formation rate based on calcein double-labeling imaging. (**H–K**) Analysis of Wnt3a and osteoblastic differentiation-related gene expression in mandibular bone, including osteocalcin, Runx2 and Osx. Data are represented as mean ± SEM (*n* = 8/group). ***P < 0.001 vs. the db/db mice group; ^###^*P* < 0.001 vs. the db/db mice with PEMF exposure group by one way analysis of variance (ANOVA) followed by Bonferroni’s multiple comparison.

Based on the micro-CT, histological and dynamic histomorphometric results, the db/db mice with in vivo Wnt3a silencing showed minor decline in mandibular trabecular bone volume fraction, cortical bone thickness, and bone formation rate, but these changes did not reach statistical significance (Fig. 7A–G). In contrast to the db/db mice with PEMF exposure, the db/db mice with PEMF exposure and Wnt3a silencing had significantly lower trabecular BV/TV, trabecular area, cortical bone thickness, MAR and BFR/BS (Fig. 7A–G, P < 0.001). Our PCR results showed that mandibular Wnt3a gene expression had remarkable reduction following in vivo Wnt3a silencing (Fig. 7H, P < 0.001). The osteogenic differentiation-related gene expression of mandibular bone (including osteocalcin, Runx2 and Osx) in db/db mice with PEMF together with Wnt3a silencing treatment exhibited significant decrease as compared with those in db/db mice with PEMF exposure (Fig. 7I–K, P < 0.001).

## Discussion

In this study, we show that: (i) PEMF exposure preserves trabecular bone architecture, increases cortical bone thickness, and improves material properties in mandibles of db/db mice; (ii) PEMF stimulates mandibular bone anabolism in vivo and improves osteoblast survival and differentiation in vitro, which are accompanied with the activation of Wnt3a/β-catenin signaling; (iii) these PEMF effects on in vitro mandibular osteoblasts are almost totally abolished after Wnt3a silencing, which are equivalent to the effects following blockade of broad-spectrum antagonist; (vi) the PEMF effects on mandibular bone quantity and quality in vivo are also abrogated after Wnt3a silencing.

The mandibular bone, responsible for chewing, speaking and supporting the lower teeth, is remodeled faster than the other skeletal sites due to metabolic and mechanical demands^40^. Compromised mandibular bone architecture and quality are observed in T2DM patients, which threaten their dental health and implant stability^7^. Consistently, our current study demonstrates that type 2 diabetic db/db mice exhibited deteriorated trabecular bone microstructure and decreased cortical bone thickness as compared to controls. Further, our nanoindentation results reveal that db/db mice had poorer tissue-level material properties in the mandibular bone. PEMF stimulation markedly increased the mandibular trabecular bone volume and number and cortical bone thickness in db/db mice, as characterized by 46% increase of BV/TV, 63% increase of Tb.N, 33% increase of Tb.Th, 30% decrease of Tb.Sp, and 49% increase of Ct.Th. In contrast, our previous study reveals similar degree of PEMF-induced increase in trabecular and cortical bone mass in femora of db/db mice^29^, as characterized by 101% increase of BV/TV, 60% increase of Tb.N, 29% increase of Tb.Th, 36% decrease of Tb.Sp, and 36% increase of Ct.Th. These findings suggest that the mandibles and femora of type 2 diabetic db/db mice have similar response capacity to exogenous PEMF stimulation. Moreover, according to the bone histomorphometric and gene expression results, we demonstrate that the PEMF effects on mandibular bone architecture and quality were associated with the enhancement of osteoblast differentiation and bone formation rather than bone resorption. Similarly, some previous studies have also revealed the anabolic response of long bone in both normal and osteoporotic animals to PEMF stimulation^16,20,26^.

Then, we isolated primary osteoblasts from the mandibles of db/db mice and studied the biological response of in vitro osteoblasts to PEMF. The similar protocol of isolation and culture of primary osteoblasts in db/db mice has also been used by other groups^41,42^. Consistent with these findings^41,42^, our results show that primary osteoblasts from the db/db mouse mandible had decreased cell viability and differentiation as compared to controls. Furthermore, PEMF exposure was found to significantly improve the cell survival, and also increase the differentiation and mineralization potential of osteoblasts from the db/db mouse mandible. Several previous studies have revealed the similar findings from normal animal calvarial osteoblasts^25,43^. Coupled with our in vivo findings, the in vitro results confirm the high sensitivity of mandibular osteoblasts of db/db mice to exogenous PEMF stimulation.

Wnt signaling plays an essential role in bone development and bone hemostasis maintenance^30,44^, and recent findings suggest that it also mediates diabetic bone deterioration^45,46^. Thus, we study the mechanism by which PEMF regulates bone quality and metabolism from the perspective of Wnt signaling. Firstly, we identified the gene expression of critical Wnt ligands in mandibles of db/db mice both in vivo and in vitro. Our results show that db/db mice exhibited decrease in the expression of canonical Wnt ligands, including Wnt1 and Wnt3a, but not in Wnt5a expression (the non-canonical Wnt ligand). Further, PEMF stimulated significant up-regulation in Wnt3a but not in Wnt1 expression in the db/db mouse mandible. PEMF-induced increase in Wnt3a but not Wnt1 or Wnt5a was further verified in primary osteoblasts from the db/db mouse mandible. Then, we examined the gene expression of key downstream molecules of Wnts. Our in vivo findings show that db/db mice exhibited significantly lower β-catenin expression in the mandible than controls, which were consistent with previous findings in long bone^35^. PEMF was found to stimulate prominent increase in β-catenin expression in the db/db mouse mandible. Similarly, our in vitro findings reveal that PEMF induced up-regulation of p-GSK-3β and β-catenin protein expression in mandibular osteoblasts from db/db mice. Thus, the current study provides strong evidence that PEMF stimulates the activation of Wnt3a/β-catenin signaling in osteoblasts of the db/db mouse mandible.

Then, we used the antagonist or gene silencing technique to identify the role of the canonical Wnt signaling in PEMF-induced enhancement of osteoblast viability and function in the db/db mouse mandible. DKK1 is known to be a secreted protein that binds to the Lrp5/6 coreceptors, causing the blockade of the interaction with all secreted Wnts and subsequent β-catenin degradation^47^. Our in vitro findings demonstrate that PEMF-induced improvement in survival and differentiation in primary osteoblasts in the db/db mouse mandible was almost totally abrogated when cells were pretreated with DKK1, highlighting the essential role of canonical Wnt signaling in PEMF effects on db/db mouse mandibular osteoblasts. Further, we used the gene silencing technique to inhibit the expression of Wnt3a subtype. We found that specific blockade of Wnt3a also abolish PEMF-induced improvement in viability and differentiation in primary osteoblasts of db/db mouse, and the degree of suppression was similar with the effects using the antagonist. Coupled with results of specific increase in Wnt3a expression in mandibles both in vivo and in vitro induced by PEMF, we conclude that Wnt3a/β-catenin signaling plays an essential role in mediating the PEMF effects on promoting osteoblast viability and differentiation in the db/db mouse mandible.

Finally, we used the gene silencing technique to block the Wnt3a expression in vivo in the db/db mouse mandible. Similar technique has also been employed to antagonize the specific target molecule of osteoblasts in vivo in previous studies^48,49^. Our PCR results reveal that mandibular Wnt3a gene expression was significantly blocked in db/db mice after treatment with Wnt3a siRNA, which further validates the effectiveness of the in vivo silencing technique. We observed that the db/db mice pretreated with Wnt3a siRNA no longer have increase in either mandibular trabecular or cortical bone mass in response to PEMF stimulation. Furthermore, PEMF-induced enhancement in mandibular bone formation and osteogenic differentiation was nearly totally abolished observed in db/db mice treated with Wnt3a siRNA. Thus, our study provides strong evidence that Wnt3a/β-catenin signaling is the key regulators of PEMF-induced improvement of mandibular bone architecture and bone anabolism in db/db mice.

Some previous studies have also investigated the changed degree of the gene expression of Wnt signaling in long bone after PEMF stimulation^50,51^. The current study found that PEMF induced highly specific upregulation of Wnt3a (but not Wnt1 or Wnt5a) and its downstream p-GSK-3β and β-catenin both in gene and in protein expression in mandibles of db/db mice both in vivo and in vitro. Moreover, these PEMF effects on mandibular osteoblasts in db/db mice were almost totally abolished after Wnt3a silencing in vitro, which were equivalent to the effects following blockade of broad-spectrum antagonist, emphasizing the importance of Wnt3a in PEMF-mediated modulation of bone formation. The PEMF effects on mandibular bone quantity and quality were also abrogated after using the in vivo Wnt3a silencing technique. In particular, no study to our knowledge has ever used in vivo silencing technique to study the mechanism by which PEMF controls bone status. The current study represents the first effort using the combination of in vitro and in vivo silencing technique to identify the role of Wnt3a in PEMF-mediated regulation of bone metabolism, which provides full evidence that Wnt3a-mediated (rather other canonical/non-canonical Wnt ligands) downstream β-catenin activation plays an essential role in PEMF-induced bone anabolism in pathological T2DM conditions.

Herein, we found the key role of Wnt3a/β-catenin signaling in PEMF-mediated modulation of bone anabolism in db/db mice, whereas the mechanism by which PEMF exposure induces specific increase in Wnt3a expression remains unclear. Previous studies have shown that PEMF exposure has the capacity of triggering dynamic changes of intracellular messenger molecules, such as calcium, nitric oxide, and cAMP^52–54^. These molecules can initiate substantial downstream signaling cascades, and thereby regulate nuclear gene transcription and protein expression. Thus, in the future study, real-time fluorescence imaging of messenger molecules combined with the high-throughput RNA-seq and Chip-seq techniques will be employed to identify the mechanism by which PEMF-mediated transcriptional regulation of Wnt3a in mandibular osteoblasts in db/db mice.

In conclusion, the current study for the first time reveals that low-intensity PEMF exposure is able to effectively improve mandibular bone mass, architecture, material properties and bone formation as well as osteoblast survival and differentiation in type 2 diabetic db/db mice, which highly depends on the activation of Wnt3a/β-catenin signaling. Our study indicates that PEMF might become a non-invasive and safe treatment alternative for resisting the deterioration of mandibular bone architecture and quality in T2DM patients, which is beneficial for protecting the teeth from loosening/loss and also securing the dental implant stability.
